# CMOST: an open-source framework for the microsimulation of colorectal cancer screening strategies

**DOI:** 10.1186/s12911-017-0458-9

**Published:** 2017-06-05

**Authors:** Meher K. Prakash, Brian Lang, Henriette Heinrich, Piero V. Valli, Peter Bauerfeind, Amnon Sonnenberg, Niko Beerenwinkel, Benjamin Misselwitz

**Affiliations:** 1 Division of Gastroenterology, University Hospital Zurich (USZ), Rämistrasse 100, 8091 Zurich, Switzerland; 2 Department of Biosystems Science and Engineering, ETH Zurich, 4058 Basel, Switzerland; 3 SIB Swiss Institute of Bioinformatics, 4058 Basel, Switzerland; 4 The Portland VA Medical Center, P3-GI, 3710 SW US Veterans Hospital Road, Portland, OR 97239 USA

**Keywords:** Colorectal cancer, Screening colonoscopies, Screening intervals, Cost-effectiveness, Microsimulation

## Abstract

**Background:**

Colorectal cancer (CRC) is a leading cause of cancer-related mortality. CRC incidence and mortality can be reduced by several screening strategies, including colonoscopy, but randomized CRC prevention trials face significant obstacles such as the need for large study populations with long follow-up. Therefore, CRC screening strategies will likely be designed and optimized based on computer simulations. Several computational microsimulation tools have been reported for estimating efficiency and cost-effectiveness of CRC prevention. However, none of these tools is publicly available. There is a need for an open source framework to answer practical questions including testing of new screening interventions and adapting findings to local conditions.

**Methods:**

We developed and implemented a new microsimulation model, Colon Modeling Open Source Tool (CMOST), for modeling the natural history of CRC, simulating the effects of CRC screening interventions, and calculating the resulting costs. CMOST facilitates automated parameter calibration against epidemiological adenoma prevalence and CRC incidence data.

**Results:**

Predictions of CMOST were highly similar compared to a large endoscopic CRC prevention study as well as predictions of existing microsimulation models. We applied CMOST to calculate the optimal timing of a screening colonoscopy. CRC incidence and mortality are reduced most efficiently by a colonoscopy between the ages of 56 and 59; while discounted life years gained (LYG) is maximal at 49–50 years. With a dwell time of 13 years, the most cost-effective screening is at 59 years, at $17,211 discounted USD per LYG. While cost-efficiency varied according to dwell time it did not influence the optimal time point of screening interventions within the tested range.

**Conclusions:**

Predictions of CMOST are highly similar compared to a randomized CRC prevention trial as well as those of other microsimulation tools. This open source tool will enable health-economics analyses in for various countries, health-care scenarios and CRC prevention strategies. CMOST is freely available under the GNU General Public License at https://gitlab.com/misselwb/CMOST

**Electronic supplementary material:**

The online version of this article (doi:10.1186/s12911-017-0458-9) contains supplementary material, which is available to authorized users.

## Background

Colorectal cancer (CRC) is the second most common deadly cancer in the United States [1] and many other Western countries. It represents an important public health burden in industrialized countries and increasingly also in developing countries [2]. Since treatment options for advanced cancer are limited, current public health strategies focus on CRC screening for prevention of mortality.

CRC is remarkable for the long-term presence of adenomatous carcinoma precursors [3, 4]. Early adenomas are, by definition, adenomas with a tubular histology and a size of less than 10 mm. Advanced adenomas are either larger than 10 mm in size or display an advanced villous or serrated histology. Adenomas are more frequent in males and their frequency increases with age. While adenomas are infrequently observed before the age of 30 years, within the screening population of 50–80 year olds, prevalence rates of at least one early adenoma and at least one advanced adenoma are 30% and 6%, respectively [5]. The presence of adenomas is an important risk factor for subsequent CRC [6].

The natural history of CRC, i.e., the sequence of events leading to CRC in each individual patient, has not been sufficiently clarified. For instance, the adenoma dwell time, i.e., the average time from initiation of an adenoma until initiation of CRC, remains unknown [7, 8]. Furthermore, several lines of evidence indicate that right sided CRC (close to cecum) and left sided CRC (close to rectum) differ regarding their biological behavior: a significant fraction of right-sided CRC derives from serrated adenomas with possible faster progression rates and lower detectability during endoscopy [9, 10].

Endoscopy is an attractive method for CRC screening because adenomas can be removed by polypectomy during this intervention, thus effectively preventing CRC [6]. Additional benefits of CRC screening include identification of high-risk individuals for surveillance via the presence of adenomas. Furthermore, screening detects CRC at earlier stages with better survival as compared to symptomatic cancer [11]. The effectiveness of rectosigmoidoscopy, which only visualizes the left-sided colon, in reducing CRC incidence and mortality was demonstrated in several large randomized controlled studies [12–16]. Colonoscopy, which visualizes the whole colon, may be similarly effective as rectosigmoidoscopy [17–20], but rigorous randomized controlled trials have been initiated only recently [21]. As such, the true magnitude of CRC risk reduction by colonoscopy is still unknown. Despite these limitations, colonoscopy screening programs have been implemented in several industrialized countries. It is generally recommended that individuals between ages 50–75 years undergo screening every 10 years, an interval which has not been determined in any systematic way [22]. Alternative CRC screening methods that have also been shown to reduce CRC mortality include detection of fecal occult blood in the stool as a cancer biomarker [23].

The optimal application of CRC screening remains a central task in gastroenterology. However, most of the open questions, such as optimal timing of multiple screening colonoscopies, will likely never be answered by high-quality clinical studies. Randomized trials of CRC screening would need a long follow-up of at least 10 years, its costs are unlikely to be covered by commercial sponsors, and patient compliance might be low due to the (largely unwarranted) perception of colonoscopy being unpleasant or dangerous. Due to these limitations, alternative strategies for studying CRC prevention using computer simulations have been developed.

In microsimulations, a large, simulated, population of many individual patients is followed throughout individual lifetimes for colonic lesions. Microsimulations account for the individual risk for colorectal cancer, the age-dependent gradual appearance of adenomas, the development of adenomas into cancer, and the detection and treatment of CRC. Assumptions about occurrence and growth rates of adenomas are calibrated to reflect the natural history of CRC. Further, the effect of screening interventions and polypectomy can be incorporated into the model. Simulation experiments are appealing, because a calibrated and validated model allows for the assessment of a wide range of medical screening interventions, such as multiple screening colonoscopies, in a time-efficient and cost-effective manner.

Three microsimulation models, namely Microsimulation Screening Analysis (MISCAN), Colorectal Cancer Simulated Population model for Incidence and Natural history (CRC-SPIN), and Simulation Model of Colorectal Cancer (SimCRC) have been described (http://cisnet.cancer.gov/colorectal/ [24]). These models have been very useful to evaluate various screening interventions for CRC with a high degree of confidence [25–29], and they share several features. All models assume that carcinomas develop exclusively from adenomas. Adenomas progress to advanced adenomas or cancer following specific rules and parameters are fit to match the epidemiologically observed prevalence of adenomas, advanced adenomas, and cancer. However, individual model predictions differ and depend on model assumptions and on the choice of parameters [8].

Adenoma dwell time, defined as the time from the appearance of clinically detectable adenoma to the appearance of carcinoma, presents the window of opportunity for effective screening. However, the value of this critical parameter remains unknown. It cannot be determined empirically since adenomas cannot be left *in situ*. The published microsimulation models assume different values: MISCAN assumes a short adenoma dwell time of 6 years, resulting in rapid adenoma progression and aggressive behavior. By contrast, SimCRC and CRC-SPIN use a dwell time of 23 and 18 years, respectively, leading to slow adenoma progression and more benign behavior [30]. Differences of MISCAN, SimCRC, and CRC-SPIN are not restricted to adenoma dwell time, and it remains unknown which model features are responsible for differences in predictions. Further, even though general features of all microsimulation models are described, the models are not publicly available and hence predictions of these models cannot be independently reproduced or advanced.

There is a growing awareness towards CRC screening and its costs and a constant need to determine the cost-effectiveness of various screening strategies. Furthermore, many new countries with different CRC epidemiology and health-care costs will be implementing CRC screening programs. Thus, there is an immediate requirement for an open source tool that is transparent, easily accessible, and adaptable for addressing highly relevant clinical and health economy questions.

Here, we present Colon Modeling Open Source Tool (CMOST), a new, open-source CRC microsimulation model. We calibrated our model against clinical data including a large rectosigmoidoscopy screening trial and compared it to existing microsimulation tools. We developed three parameterizations of our model differing in adenoma dwell time. Our results show that adenoma dwell time influences some but not all predictions of our CRC microsimulation model. The model offers the flexibility to design new screening protocols and costs as well as to reparameterize for the natural history by working with the automated calibration.

## Methods

### Characteristics of the CMOST microsimulation model

**Fig. 1:**
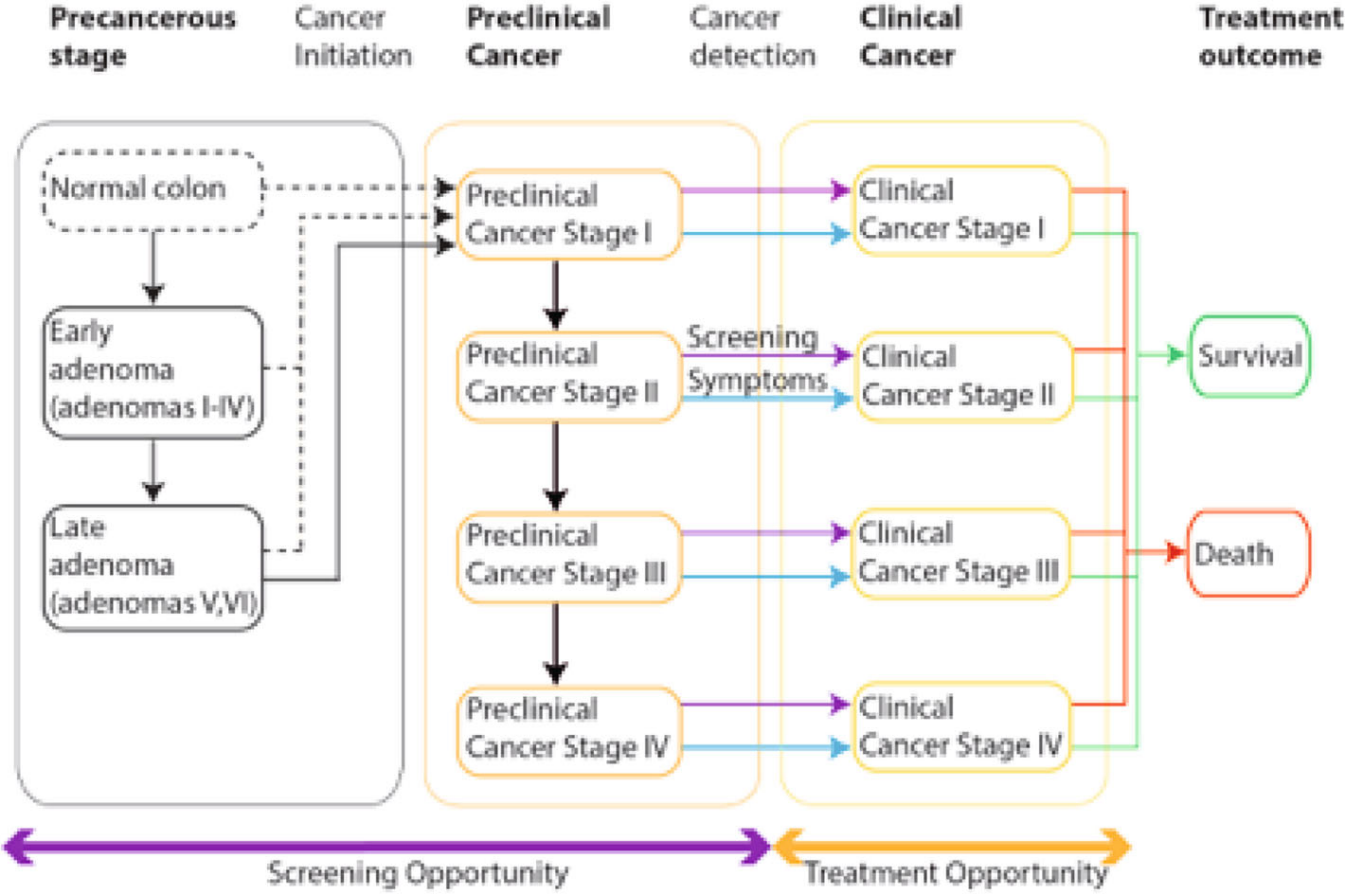
Structure of the microsimulation model used in CMOST. Most cancers in CMOST start as adenomas (stages I to VI) and progress to cancer. Adenomas may be diagnosed and removed by screening interventions. Few cancers appear directly without adenoma precursors. Preclinical cancer can be diagnosed at an early stage by screening. Cancer develops in four stages. It can be detected by screening or due to symptoms. After detection, treatment can cure cancer with a stage-dependent probability

We developed a new microsimulation model for CRC, focusing on the natural history as observed by endoscopy on a macroscopic and histological level. The model has been implemented in Matlab as the program Colon Modeling Open Simulation Tool (CMOST). In our model, colorectal cancer may develop either through adenomatous precursors detected by colonoscopy or in a non-adenomatous pathway. Once an adenoma has been transformed into cancer, it can be detected in an asymptomatic state during diagnostic examinations such as endoscopy or when it becomes symptomatic (Fig. 1). The pseudocode illustrating how our CMOST microsimulation program works is given in Table 1.

**Table 1:** Pseudocode for the CMOST program flow implemented in MATLAB

MAIN MODULE OF CMOST: individually tracks every individual in a population
Input:
- Model parameters determining natural history of CRC
- Settings for CRC screening and surveillance
Output:
- Death year, cause of death and years lost to CRC for each individual
- CRC incidence, prevalence, stages, mode of detection and outcome for each CRC and each individual
- Adenoma prevalence and adenoma stages for the whole population
- Usage of screening and surveillance interventions
- All CRC related costs
for Year = 1 to 100 and if Alive = ‘YES’
for IndividualNumber = 1 to NumberOfIndividuals
call function AdenomaNaturalHistory(Record)
%--- updates the natural history of adenomas and development to cancer
if (Screening.Mode=’ON’ and Screening.Preference=’COLONOSCOPY’ and Screening.Year=Year) or
(Cancer.Symptomatic=’YES’) or (FollowUp.Year = Year)
%--- if colonoscopy is a preferred screening option, or if cancer is symptomatic or for followup
call function Colonoscopy
if Treatment=’ON’ and (Year-Cancer.DetectionYear) > 5 years
Treatment=’OFF’
elseif Treatment=’ON’ and (Year-Cancer.DetectionYear) < 5 years
call function AddCost(Treatment=’ON’)
if rand > DeathFromCancer.Chance
Alive.Cancer=’NO’
DeathFromCancer.Year = Year % --- used for treatment costs in the last year before death from cancer
elseif rand > DeathFromNaturalCauses.Chance
Alive=’NO’
end IndividualNumber
end Year
function AdenomaNaturalHistory(Record)
% --- new adenoma(s) appear (influenced by age, gender, colon location, individual risk)
Record.(AdenomaStage=I) = Record.(AdenomaStage=I) + ChanceOfNewAdenoma
% --- adenoma progression (influenced by age, gender, colon location, individual risk)
Record.(AdenomaStage=II to VI) = Record.(AdenomaStage=II to VI) + AdenomaStageProgression
Record.CancerStage(1) = Record.CancerStage(I) + AdenomatousCancer(FromAdenomaStage=VI)
+ FastCancer(FromAdenomaStage=I to V) + DirectCancer(FromNoAdenoma)
Record.CancerStage(2 to 4) = Record.CancerStage(2 to 4) + CancerStageProgression
Cancer.Symptomatic = ChanceOfSymptoms(Record.CancerStage)
end function AdenomaNaturalHistory
function Colonoscopy(Record)
Adenoma.Detected = ChanceOfAdenomaDetection(Record.AdenomaStage, Record.AdenomaLocation)
Cancer.Detected = ChanceOfCancerDetection(Record.CancerStage, Record.CancerLocation)
if Cancer.Detected = ’YES’
Cancer.DetectionYear = Year
Record.Cancer = 0%--- assume curative treatment has been performed
Treatment = ’ON’
elseif Adenoma.Detected = ’Yes’
Polypectomy = ’YES’
Record.Adenoma = 0%--- remove the adenomas by polypectomy
Adenoma.Detected = 0
FollowUp.Year = RecommendedYear % --- recommend followup according to guidelines
if rand > ChanceOfComplication
Complications = ’YES’
If rand > ChanceOfDeathFromComplications
Alive = ’NO’
call function AddCosts(Colonoscopy, Polypectomy, Complications)
end function Colonoscopy
function AddCosts
Cost = Cost + if(Colonoscopy = ’YES’) x InputCost.Colonoscopy +
and in the last year of death from cancerif(Polypectomy = ’YES’) x InputCost.Polypectomy +
and in the last year of death from cancerif(Complications = ’YES’) x InputCost.Complications +
and in the last year of death from cancerif(Treatment = ’ON’) x InputCost.TreatmentCost(Year – Cancer.DetectionYear)
and in the last year of death from cancer%--- the treatment cost is divided into 3 stages: initial, continuing and last year costs for the first quarter, upto 5 years and in the last year of death from cancer
end function AddCosts

**Fig. 2:**
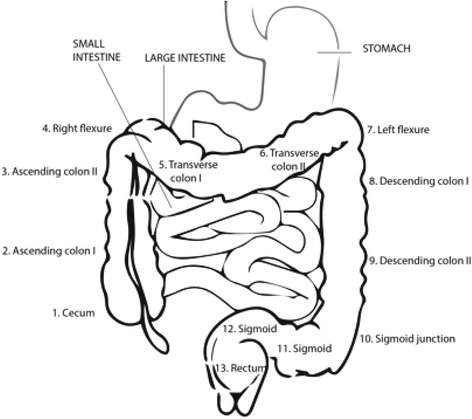
Location of adenomas and cancer within the colon. The large intestine has been divided into 13 relevant segments. The CMOST model assumes location dependence for the progression and detectability of adenomas and cancers. Colonoscopy reaches the cecum in 95% of cases. Rectosigmoidoscopy is easier to perform but limited in scope and meant to reach the left flexure in the majority of cases

In our model, an adenoma develops within one of 13 colon segments (Fig. 2). This location remains constant for the lifetime of a lesion affecting both rate of progression as well as accessibility and detection by endoscopic methods such as rectosigmoidoscopy and colonoscopy. Altogether 6 distinct adenoma stages are distinguished: Stages I-IV correspond to early adenomas with sizes of 3 mm, 5 mm, 7 mm, and 9 mm, respectively. Stage V corresponds to an adenoma ≥1 cm or an adenoma with advanced villous histology, and stage VI corresponds to an adenoma of size >2 cm.

CMOST tracks the history of a general population from birth until death or a maximum age of 100 years. Adenoma initiation, progression to advanced adenoma and cancer, cancer progression, screening, and surveillance are all modeled in time increments of 3 months.

### Choice of parameters

The relevant parameters used for the description of the natural history of CRC are summarized in Table 2, and their values are provided in the supplementary information. Some of these parameters depend on age and hence are defined by a function, for instance the age-dependent adenoma initiation risk of an individual is assumed to be a sigmoidal function. The sigmoid function was chosen since the benchmark parameter (i.e. age-dependent adenoma prevalence) also follows a sigmoidal function and can thus be conveniently reproduced. The model parameters age dependent early/advanced adenoma progression are described by Gaussian functions, since these functions provide maximal flexibility allowing for a monotonously increasing or decreasing curve or a curve with a maximum.

**Table 2:** Description of the different parameters used in CMOST model. The procedure for calculating the parameters is described in the supplementary information

Model parameter	Properties	Functional form (where applicable)
Age-dependent adenoma initiation risk	Defined by a sigmoidal function.	$\frac{a_0}{1 + \exp \left(-\left({a}_1 y-{a}_2\right)\right)}$
Age-dependent early adenoma progression risk	Defined by a Gaussian function.	*b*_0_ exp(−(*b*_1_ *y* − *b*_2_)^2^)
Age-dependent advanced adenoma progression risk	Defined by a Gaussian function.	*c*_0_ exp(−(*c*_1_ *y* − *c*_2_)^2^)
Individual adenoma risk	Cumulative density function describing the relationship between population proportions and individual risk for appearance of new adenomas. Defined by automatically calibrated anchor points.	
Early and advanced adenoma specific progression risk	Cumulative density function describing the relationship between early or advanced adenoma proportions and adenoma specific progression risk. Defined by manually calibrated anchor points.	
Correction factors male/female for i) adenoma initiation ii) early adenoma progression iii) advanced adenoma progression	Constant	
Correction factor rectum/colon for i) early adenoma progression ii) advanced adenoma progression	Constant	
Adenoma stage-specific progression risk	Constant. One value for each adenoma stage 1-6, enables fine tuning of the distribution of adenoma stages 1-6	
Adenoma stage-specific regression risk	Constant. One value for each adenoma stage (1-6 highest for 1, lowest for 6); not calibrated	
Adenoma stage-specific cancer risk	Constant. One value for each adenoma stage (1 to 5), calibrated to match prevalence of cancer in adenomas	
Location specific direct cancer risk	Constant. One value for each colon segment 1-13 (calibrated to match CRC incidence reduction in rectosigmoidoscopy CRC prevention study)	

Other model parameters are assumed to be stochastic and hence are defined by an assumed probability distribution. In this case the value for the individual or the adenoma is drawn randomly from the respective distribution in each simulation run. The parameter will then remain constant throughout the lifetime of the individual or adenoma. Examples include the risk for new adenomas specific for an individual or a progression risk specific for a given adenoma (see below). Other parameters, e.g., the relative risk of males versus females, are scaling factors for the respective condition.

Please note that our parameters cannot be directly compared to parameters from existing microsimulation models since different strategies for the description of the natural history of CRC were used [31]: For MISCAN, the duration of an adenoma in a given state (not adenoma progression rates) are used to describe the natural history of CRC and duration of adenomas are drawn from an exponential distribution. For CRC-Spin, adenoma growth follows a Janoschek growth model and CRC growth an exponential model. Finally, in SimCRC describing adenoma initiation and progression follow logistic regression curves, resembling our description of the natural history of CRC closest. It should be noted that due to the limited understanding of the natural history of CRC, the best mathematical descriptions of adenoma and CRC initiation and progression cannot be determined.

### Simulating the natural history of CRC

At birth, an individual adenoma risk is assigned to each individual, which defines high or low CRC risk for this individual. At each time increment we decide about initiation of an adenoma, at random, considering both the individual adenoma risk and the age dependent adenoma risk. Upon adenoma initiation, an adenoma specific progression risk will be assigned to each adenoma. A high risk defines a quickly progressing aggressive adenoma, whereas a low risk defines a benign adenoma.

At each time increment, each adenoma might undergo one of the following changes: i) Adenoma progression: the risk for progression is determined by the product of an age-dependent adenoma progression risk, the progression risk of the given adenoma, the progression risk of the adenoma stage, and correction factors for female gender or rectal location if applicable. ii) Fast progression of adenoma to cancer: the risk of this event depends on the age-dependent advanced adenoma progression rate, the stage of the adenoma, gender, and location. iii) Adenoma regression: with very low probability, regression of an adenoma (or disappearance of a stage-I adenoma) is possible [32–36]; the risk of this event is defined by the adenoma stage.

Initiation of CRC can happen via three different pathways (Fig. 1): i) Progression of a stage 6 adenoma: this pathway accounts for approximately two thirds of all carcinoma in our microsimulation. ii) Fast progression of a smaller adenoma (stage 1-4) as described above. iii) Direct cancer without adenomatous precursors; such a pathway was introduced to match data from randomized endoscopic CRC prevention trials [12, 15, 37, 38] regarding prevention of right-sided adenomas after rectosigmoidoscopy screening. This direct cancer pathway represents cancers with precursors difficult or impossible to detect by colonoscopy. These cancers occur preferentially within the right colon and the direct cancer pathway reflects the reduced efficacy of CRC prevention by colonoscopy regarding right-sided CRC [39, 40].

At initiation of a cancer a sojourn time, i.e., the time from the initiation of the cancer to it becoming symptomatic, is assigned to the cancer. The sojourn time is drawn from a normal distribution of values with a mean of 3 years [8, 41] and a standard deviation of 0.5 years. The stage at which symptoms will appear is chosen to reflect the stage distribution of symptomatic cancers. The time spent in each stage is also predefined for each cancer to reflect the stage distribution of cancers detected during colonoscopy screening [42].

After sojourn time a carcinoma will become symptomatic, triggering a diagnostic colonoscopy. Upon detection of a cancer, survival time will be defined, following published survival rates for the respective CRC stage [43]. Death from CRC is restricted to 5 years after CRC diagnosis.

In our microsimulation death results from colon cancer, medical interventions, or from other age-dependent causes of mortality according to the 2008 US Life Table Data [44]. An individual patient who dies of CRC is followed in the simulation until he would have died from other causes.

### Simulation of CRC screening

CMOST supports simulation of CRC screening and detection by colonoscopy. For each colonoscopy, the amount of colon visualized is drawn from a specified distribution, with 95% of all colonoscopies reaching the cecum on average. For each adenoma, our microsimulation will determine whether the respective lesion will be detected during a given intervention [45]. The probability of detection depends on adenoma stage and whether the lesion resided within the visualized colon. CMOST also accounts for slightly reduced adenoma detection at the hepatic and splenic flexure. CMOST considers the following complications of colonoscopy: i) major bleeding (4 per 10,000), ii) minor bleeding (11 per 10,000), iii) mucosal burn (3 per 10,000) and iv) perforation (7 per 10,000) [46]. The probabilities of these complications are derived from published data and increase 2-fold after polypectomy [47].

Rectosigmoidoscopy is implemented similar to colonoscopy but with the limitation that the colon is visualized only from rectum to the left-flexure (on average 6 of the 13 segments of the colon). Due to poor bowel preparation and additional technical limitations, the probability to detect an adenoma is assumed to be lower than for colonoscopy (12.5% lower probability of detection for advanced adenomas and 25% for early adenomas compared to colonoscopy). The risk of complications during rectosigmoidoscopy is much lower than for colonoscopy [48] and no lesions will be removed. Any lesion detected at this intervention will trigger a colonoscopy.

CMOST also supports additional screening interventions including fecal occult blood test (FOBT) or immune -FOBT. The parameters of all screening intervention as well as adherence to screening and follow-up colonoscopy can be freely adjusted. CMOST allows for the definition of a screening plan with a starting date, a finishing date, an adherence rate and a screening interval.

### Cost calculations

Our model includes costs for screening colonoscopy, potential polypectomy, and procedural complications. In our cost calculations, treatment costs during 5 years after cancer diagnosis are considered. Treatment is divided into three phases: The initial care phase lasting 3 months includes cancer detection, surgery and radiation as well as chemotherapy in selected situations depending on the stage and location of the carcinoma. In case of death from CRC, the phase of terminal care lasts up to 12 months and includes additional palliative surgery in 50% and palliative chemotherapy in all patients. The period of continued treatment with monitoring and consultation visits spans from the end of the initial phase to the beginning of the last year or the end of treatment. Screening and treatment costs (Additional file 1: Table S1) are based on the Diagnosis Related Group (DRG) codes for the outpatient setting and Current Procedural Terminology (CPT) codes for the inpatient setting, using the corresponding Medicare reimbursement schedule (http://www.cms.gov). Average national US payments for the year 2012 are used in the present calculations. Costs and life years gained are computed via comparison to those that accrue in the non-screening scenario. Both, costs and LYG are discounted by a 3% annual discount rate. A list of costs used for our study is provided in Additional file 2: Tables S7 and Additional file 3: Table S8.

### Automated parameter calibration

CMOST supports automated calibration of model parameters to meet epidemiological benchmarks regarding the natural history of colorectal cancer. To calibrate our model to the US American population, we used published age- and sex-specific adenoma prevalence rates as benchmarks [18, 49–51]. Carcinoma incidence and mortality, as well as separate colon and rectum cancer incidence rates, are modeled according to data from the Surveillance Epidemiology and End Results (SEER) database for 2005–2009 [52]. The stage distributions of symptomatic and asymptomatic cancers also follow published analyses [11, 53]. Altogether we used 105 benchmarking data points covering various quantities predicted by the model for parameter estimation. A full list of all benchmarks is provided in Additional file 1: Table S1. Our strategy for automated parameter calibration is explained in detail in Section V of the Supplement. Briefly: we divide the natural history of CRC into four steps (I: early adenoma, II: advanced adenoma, III: cancer, IV: direct cancer) and perform calibration of the group of parameters relevant for each of these steps sequentially. Our implementation of the natural history of CRC has been designed in a way that for each calibration step a lower step will influence the readouts of all higher steps but not vice versa. For instance, adjusting parameters in step 1 to increase the incidence of early adenomas by 10% will also change the incidence of advanced adenomas by 10%. Vice versa, modifying parameters of step 2 to increase adenoma progression to increase advanced adenoma incidence by 10% will affect early adenoma prevalence to a lesser degree: Faster early adenoma progression will decrease early adenoma prevalence since some early adenomas will be advanced adenomas now. However, the decrease will only be approximately 1% since prevalence of early adenomas is 10 times higher than for advanced adenomas. For these reasons, all four steps can be considered independent calibration steps and are performed sequentially and sequential calibrations yields in satisfactory results.

However, practical tests showed that even better fitting could be achieved by a final calibration step which simultaneously readjust parameters for step 2 and 3 simultaneously. Furthermore, our program provides the option to automatically perform parameter calibration of steps 1-3 sequentially, followed by a fine-tuning of parameters in steps 2 and 3 as a single procedure (for instance on a cluster computer). Details of these procedures are explained in the Manual.

For each calibration, we used a heuristic greedy algorithm followed by Nelder-Mead optimization [54] to minimize the squared error between the benchmark values and the corresponding model predictions for parameter estimation. Automated parameter calibration is required only when the benchmarks of the natural history are changed or when different possibilities for the adenoma dwell time within the same natural history are to be explored.

### Implementation of CMOST

Details of all features of CMOST can be found in the accompanying manual. The software is available under a GNU general public license and can be downloaded at https://gitlab.com/cmostmodel/CMOST. CMOST has been implemented in Matlab®. Time critical calculations were implemented using the Coder module of Matlab, resulting in a 20x improvement in performance.

All functionality of CMOST is available via several intuitive graphical user interfaces. All relevant parameters, including values for all variables describing the natural history, specifications for CRC screening, and a screening plan, can be saved and loaded as a settings file. CMOST can be run on a desktop computer and we provide basic support in the form of scripts for running it on LINUX compute clusters.

CMOST produces the following output files after each calculation: i) a Matlab data file with the raw computation results, which can be used for further calculations in Matlab; ii) several PDF files containing plots of all relevant variables that describe the prevalence and distribution of adenomas, advanced adenomas, and CRC, relative to the benchmarks used; iii) an Excel file with a summary of the CMOST simulation run.

The accurate determination of the optimal age for reduction in incidence, mortality, or cost per life year gained required calculations on population sizes of up to 10 million. The calculations are repeated for each condition, e.g., the year of recommended screening colonoscopy. Each of these cases is, in principle, a single processor job. However, in order to effectively handle the memory available on each processor, the calculation on the population of 10 million was subdivided into calculations with 100,000 individuals on a high-performance LINUX computer cluster.

## Results

### A new microsimulation model for the natural history of CRC

We developed CMOST, a microsimulation model to simulate CRC progression and the effect of CRC screening (Fig. 1). In our model carcinoma develops via early and advanced adenoma precursors. Altogether, 6 adenoma stages and 4 carcinoma stages are considered in the model. Most of the transition towards pre-clinical cancer occurs via the adenomatous pathway, advancing through the 6 successive stages of adenoma progression. With lower probability, pre-clinical cancer can also start from any of the adenoma stages or even from seemingly normal colon mucosa.

Our model accounts for the gender- and age-dependent risks of adenoma development [5, 18, 50, 55] as well as the presence of multiple adenomas. Each adenoma will be assigned one of 13 locations within the colon, reflecting the distribution of adenoma lesions within the colon [24]. In our model, rectum adenomas progress faster compared to colon adenomas to achieve the expected proportion of rectum cancers of all CRC [56]. Each individual within the simulated patient population is assigned an individual adenoma risk. The distribution of these risks within the whole population is calibrated to match the frequency of multiple adenomas [49] and also matches the frequency of synchronous colorectal cancers of 3.5% of all CRC [57].

Adenoma dwell time indicates the time between appearance of an adenoma and transition to colorectal cancer. Since adenoma dwell time can only be estimated empirically with a broad margin of error, we considered three parameterizations of CMOST with dwell times of 8, 13, and 19 years, referred to as CMOST8, CMOST13, and CMOST19, respectively.

### Automated calibration of our model

**Fig. 3:**
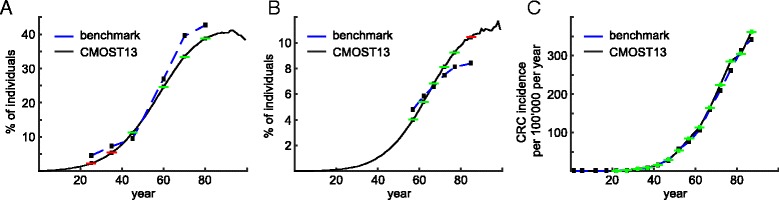
Results of CMOST regarding adenoma and cancer epidemiology for the whole population (males and females). **a**: Early adenoma prevalence **b**: Advanced adenoma prevalence **c**: Cancer incidence. Literature-derived benchmarks (see Additional file 4) used for our microsimulation are indicated by black squares and a blue dashed line; results of CMOST13 calculations are shown as a black line and green-squares when within 20% of benchmarks, and as red-squares otherwise

To calibrate our model for the North American or a similar Western population, published age- and sex-specific adenoma prevalence rates were used [18, 50, 55]. Carcinoma incidence and mortality, as well as separate colon and rectum cancer incidence rates, are modeled according to the data from the Surveillance Epidemiology and End Results (SEER) data base for 2005–2009 [52]. Altogether, 105 benchmarks were selected (Additional file 1: Table S1) and an automated calibration strategy was employed (see Additional file 4). A comparison with the benchmarks is performed at every iteration of the automated parameter calibration. The calibration is terminated when the comparisons are satisfactory, such as shown in Fig. 3. CMOST allows the flexibility of including a new set of CRC natural history benchmarks from a new population, and the four-steps of the automated parameter calibration can be repeated.

CMOST assumes a fraction of all cancers to appear without adenomatous precursors (referred to as direct cancer henceforward). Benchmarking for direct cancer was performed in an indirect way using data from a randomized rectosigmoidoscopy study with 170,432 individuals between 55 and 64 years of age [58]. After a one-time only rectosigmoidoscopy (visualizing the left-sided colon), individuals with positive findings are followed by a full colonoscopy, thus enabling CRC prevention on the left and the right side of the colon. Overall CRC incidence reduction was used as a benchmark to parameterize the rate of direct cancers (further details in Additional file 4).

### Simulation of CRC screening

In our microsimulation, colonoscopy will either be performed according to a pre-defined screening schedule, for adenoma or cancer surveillance, or for the diagnosis of symptomatic cancer. Our implementation of colonoscopy accounts for varying probabilities of successful visualization of the hepatic and splenic flexures and imperfect detection of early and advanced adenomas and colorectal cancer [45, 59]. Complications of colonoscopy including major bleeding, which requires hospitalization; minor bleeding, which does not require hospitalization; perforation, which requires surgical treatment; and mucosal burn, are all accounted for [60]. We also simulated surveillance colonoscopies after the detection of a lesion–according to current guidelines [61]. Other screening interventions, including rectosigmoidoscopy and fecal occult blood tests with various test parameters, have also been implemented. Our program can be used to assess effects of every CRC screening method including DNA stool tests, DNA blood tests or imaging based tests provided that information about test sensitivity, specificity, risks and the costs are available.

### Comparison of CMOST to randomized CRC screening trials

To validate CMOST, we compared the predictions of our model to the independent results of a large randomized controlled trial of endoscopy for CRC prevention [37] (Table 3) that were not used for model calibration. In this study, the effect of one or two rectosigmoidoscopies in a combined study group of 77,445 individuals aged 55-74 years was tested over an 11.9-year follow-up period. While the study is similar to the one used for benchmarking the direct cancers [12], the study group is slightly larger, start and end times differ, and two endoscopies are used in a significant fraction of patients. Results of CMOST13 simulations compare well with the intention-to-treat analysis of the randomized study and its confidence intervals: CMOST13 predicts a similar overall incidence reduction (19.9% predicted vs. 21% observed), left-sided CRC incidence reduction (24% predicted vs. 29% observed) and overall mortality reduction (26% predicted vs. 26% observed) [37]. Similar results were obtained using CMOST8 and CMOST19 (Table 3). We also simulated other smaller CRC prevention studies (Additional file 5: Table S9), and the simulation results were also in agreement with the observed data [15, 38]. We conclude that CMOST reflects the natural history of CRC well and reliably predicts the outcome of randomized rectosigmoidoscopy screening trials.

**Table 3:** Effects of rectosigmoidoscopy screening for CRC prevention combined with 11.9-year follow-up (intention to treat analysis according to the randomized controlled study by Schoen et al. [37])

		Schoen et al. [37] with 95% CI	CMOST8	CMOST13	CMOST19
Incidence reduction	All CRC	21% (28-15)	20%	20%	20%
	Right-sided CRC	14% (3-24%).	15%	7%	5%
	Left-sided CRC	29% (20-36).	21%	24%	26%
Mortality reduction		26% (13-37)	23%	26%	23%

### Comparison of CMOST with existing CRC simulation models

**Fig. 4:**
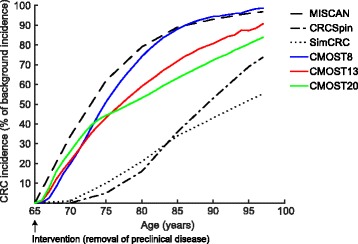
Maximum clinical incidence reduction: The incidence of cancer following a hypothetical perfect intervention which removes all adenomas and undiagnosed cancers at age 65 is used to compare the predictions of microsimulation models. MISCAN, CRC-SPIN, and SimCRC data are from reference [7]

Predictions of CMOST were compared to those of other microsimulation models describing the natural history of CRC, namely MISCAN, CRC-SPIN, and SimCRC, following two previously published comparative studies [7, 62]. In a first comparison, a hypothetical perfect screening intervention, removing all lesions within the colon of each individual (early adenomas, advanced adenomas, and cancers) at the age of 65 was modeled (referred to as maximum clinical incidence reduction in the original publication [7]). All CRC detected after the age of 65 would thus be newly developed lesions. CMOST8, CMOST13, and CMOST19 differ slightly regarding future CRC incidence (Fig. 4). Predictions of CMOST8 strongly resemble predictions of MISCAN while CMOST13 and CMOST19 predict a lower CRC incidence 20 years after the hypothetical intervention (Fig. 4). Additional detailed comparisons between all microsimulation models are provided in Additional file 6 Tables S2, Additional file 7: Table S3, Additional file 8: Table S4, Additional file 9: Table S5 and Additional file 10: Table S6.

We also tested predictions of CMOST with regard to the effects of colonoscopy on colon cancer incidence and mortality (Table 4) and found that CMOST was closest to the MISCAN model. Since for all versions of CMOST, i.e., CMOST8, CMOST13, and CMOST19 the direct cancer rate was calibrated to achieve an identical incidence reduction as a randomized rectosigmoidoscopy study, predictions of all CMOST versions were very similar. Thereby, the fraction of direct cancer is lowest for CMOST8, intermediate for CMOST13, and highest for CMOST19. Effects of a longer dwell time (19 years for CMOST19, 13 years for CMOST13, leading to longer lasting effects of adenoma removal than for CMOST8) will be offset by a higher fraction of direct cancers which cannot be prevented by CRC screening. Without this calibration step (with identical direct cancer rates) incidence reduction for CMOST19 would be highest, intermediate for CMOST13, and lowest for CMOST8 (data not shown).

**Table 4:** Comparison of CMOST with other microsimulation tools. Screening colonoscopies were performed at 10-year intervals between ages 50 and 75 years. Results of the microsimulation tools MISCAN, CRC-SPIN, and SimCRC were taken from reference [7]. Numbers indicate percentage reduction or the number of additional colonoscopies or CRC cases as indicated per 1000 individuals. For CMOST, the numbers of the baseline scenario assuming a dwell time of 13 years (CMOST13) are given; numbers in parenthesis indicate results obtained by using CMOST8 and CMOST19

	MISCAN	CRC-SPIN	SimCRC	CMOST13 (CMOST8, CMOST19)
Incidence reduction	52%	91%	82%	53% (55, 48)
Mortality reduction	65%	92%	84%	61% (65, 55)
Life years gained	207	260	327	142 (155, 121)
Screening colonoscopies	2288	2580	2574	2373 (2304, 2289)
Surveillance colonoscopies	1715	1341	1609	1311 (1222, 1251)
Total colonoscopies	4002	3921	4184	3600 (3538, 3558)
CRC cases prevented	30	56	54	27 (28, 23)
CRC mortality cases prevented	19	25	30	12 (13, 10)
Colonoscopies per case prevented	135	70	77	132 (124, 155)
Colonoscopies per LYG	19	15	13	25 (22, 29)

On a different note, CMOST was calibrated to reflect contemporary CRC incidence, whereas MISCAN, CRC-SPIN, and SimCRC reflect the higher CRC incidence of 1977 (before the onset of CRC screening). This difference explains some of the discrepancies within Table 4 (compare “CRC cases prevented” or “colonoscopies per life year gained” for CMOST and the established models). We also simulated the effect of FOBT screening regarding CRC incidence and mortality reduction. The results obtained by CMOST are similar to those from SimCRC and MISCAN according to published data [63] (Additional file 9: Table S5, Additional file 10 Table S6). Taken together, predictions by CMOST regarding the natural history of CRC development and CRC screening are comparable to published microsimulation tools. All versions of CMOST resulted in similar predictions for various read outs, and predictions of CMOST strongly resemble those of MISCAN.

### Defining the optimal time point for colonoscopy screening

**Fig. 5:**
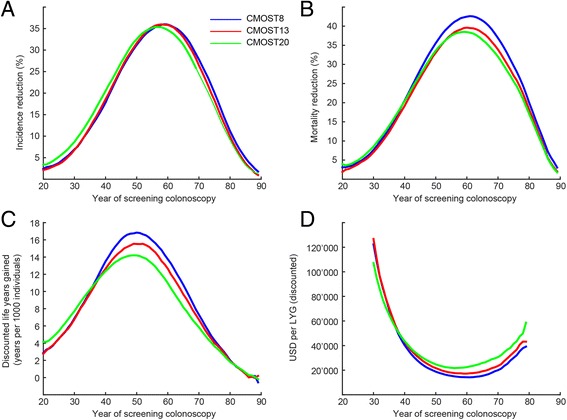
Results of the effect of a single screening colonoscopy is performed at a specific age: **a**: Incidence reduction **b**: Mortality reduction **c**: Discounted life years gained **d**: Discounted costs per discounted life year gained

We tested the optimal application of colonoscopy in a scenario allowing for a single screening colonoscopy during the lifetime of a patient. We compared incidence and mortality of CRC to a scenario without screening (Fig. 5a, b). For all versions of CMOST, incidence reduction from colonoscopy increases with age at first colonoscopy up to an optimum at approximately 58 years. Both the extent of the benefit conferred by colonoscopy (incidence reductions in CMOST8, CMOST13, CMOST19 are 36%, 36%, and 35%, respectively) and the optimal time point for colonoscopy were comparable for all simulation models tested (59, 58, and 56 years; Fig. 5a). Similarly, the mortality reductions predicted by CMOST8, CMOST13, and CMOST19 are 43%, 40%, and 39%, respectively, all at the optimal ages of 61, 60, and 50 years (Fig. 5b).

We also calculated life years gained (LYG) and cost-efficiency of colonoscopy. Following current practice in health economics, LYG and costs were compared to the standard scenario without screening. Differences were discounted by 3% per year (Fig. 5c). Since prevention of a carcinoma in a young patient will save more life years compared to an older individual, the optimum for LYG is at a lower age than for incidence and mortality reduction. For CMOST8, CMOST13, and CMOST19, 17, 16 and 14 discounted years per 1000 persons, respectively, were obtained. An optimum was reached at similar time points, (age 50, 49, and 49 years, respectively). Without discounting, the number of LYG would be considerably higher (102, 94, and 85 years per 1000 individuals) at somewhat later time points (53, 54 and 53 years, respectively).

Cost efficiency was estimated by dividing discounted costs in USD by discounted LYG. By common conventions in health economics, any intervention with costs less than 100,000 USD per LYG is considered cost-effective. In our calculations, the optimal values of cost-efficiency for a single screening colonoscopy were $14,209, $17,211, and $21,698 USD/LYG, and thus cost-effective for all three versions of CMOST. As for incidence and mortality reduction, the optimum cost-effectiveness is attained at a similar age in all versions of CMOST tested, i.e., for CMOST8, CMOST13, and CMOST19, at 61, 59, and 56 years, respectively (Fig. 5d).

## Discussion

We have introduced CMOST, a new microsimulation model for the natural history of CRC. CMOST reflects all basic characteristics of the natural history of CRC, shows comparable characteristics to published models of CRC microsimulation, and can reproduce the results of a large randomized endoscopic screening intervention.

Our tool offers several benefits to the user. First, our model is publicly available under a GNU General Public License. This will enable independent reproduction of predictions and advancement of the model and its implementation. Furthermore, publication of all details of our microsimulation will enable scrutiny and a detailed discussion regarding all relevant aspects of CRC simulations. We hope that future extensions of CMOST will help increasing the validity of simulation results and further improve the *in silico* design of CRC screening strategies. Additional advancements of our model and its implementation include calibration against results of a recently published endoscopic screening trial, which had not been possible at the time of the development of previous models. The transparency of benchmarking and automated calibration of CMOST parameters will facilitate further improvements to the model. Repeated convergence to the same set of parameters for different initial parameter guesses suggests a well-defined optimum in parameter space of CMOST.

We believe that many aspects of the CMOST implementation will benefit future users of the tool. CMOST can be run using a graphical user interface, which allows adjustment of all relevant parameters. Time critical routines are accelerated using the coder option of Matlab, with a 20x improvement in performance. A simulation of 100,000 individuals implemented in Matlab R2015 and executed on a 2.5 GHz processor, uses 1 GB RAM and typically finishes in less than 1 min. In addition, CMOST supports usage of a high-performance compute cluster for either the simulation of large populations or detailed explorations. There is no agreement as to what level of detail is necessary for a CRC microsimulation to be both accurate and practical. We attempted to implement CMOST as a detailed microsimulation. For instance, 13 different locations of a lesion within the colon are supported and many details of CRC pathogenesis and screening can be adjusted according to these locations.

Since the true value (or probability distribution) of adenoma dwell times is unknown, we calibrated versions of CMOST with median dwell times of 8, 13, and 19 years (CMOST8, CMOST13, CMOST19). Predictions of CMOST8, CMOST13, and CMOST19 were similar and showed resemblance to MISCAN. Due to our calibration process, the longer dwell time of CMOST19 (leading to longer lasting effects of adenoma removal) would be offset by a higher rate of direct cancer which cannot be prevented by endoscopic screening. As in previous studies, when MISCAN, CRC-SPIN, and SimCRC were frequently tested in parallel to increase robustness of predictions, different versions of CMOST can be run for increased robustness of predictions. Our data confirm adenoma dwell time as a critical parameter for the natural history of CRC; however, we demonstrate that after calibration for relevant clinical endpoints, similar results can be achieved with models using considerably different dwell times.

Currently available epidemiological data do not allow for distinguishing whether CMOST8, CMOST13, or CMOST19 reflects the natural history of CRC best. We were unable to calibrate models with less than 8 years of adenoma dwell time suggest that this parameter is bounded, at least for a given set of benchmarks. Predictions of all CMOST versions regarding overall CRC incidence and mortality reduction and protection from left-sided CRC were well within the confidence interval of a large endoscopic CRC prevention trial. In the future, results of colonoscopy CRC prevention trials will further inform microsimulation models regarding the prevention of right-sided CRC.

According to the standard health economic definitions, an intervention is considered cost-effective if the costs remain below 100,000 USD per life year gained [64, 65]. Colonoscopy screening in that sense is a cost-effective intervention. In agreement with the results of numerous previous microsimulations [66, 67], screening costs of a single screening colonoscopy remains below the accepted limit of 100,000 USD per life years gained. Our results also largely agree with an earlier study based on a Markov model, recommending a single colonoscopy at age 60 years as a highly cost-effective screening strategy [68]. Several previous computational studies reported an even better cost-effectiveness than our study, and some studies even predicted CRC screening to be cost saving [66]. These discrepancies are largely due to different cost assumptions. Past studies used Medicare data from the years 1999-2003 [46, 69] with some studies even considering the cost of death due to reasons other than cancer. In contrast, the cost assumptions in the present study were based on the Medicare reimbursement schedule from 2012. Therefore, in previous microsimulations screening tended to be cheaper or treatment costs were higher compared to ours, making our cost calculations conservative.

Our tool provides flexibility to easily implement and evaluate basically all CRC screening options provided information regarding test sensitivity, specificity, risks and costs are provided. Examples include DNA based serum and stool tests or imaging based screening methods. Furthermore, big efforts are currently underway to ensure the quality of colonoscopy as the most frequently used CRC screening tools: Modifications in the procedure such as endoscope inversion in the cecum to improve adenoma detection there, adjustments in bowel preparation to improve “cleanliness” of the colon, enforcing a retraction time of colonoscopy of at least 6 min have been shown to increase adenoma detection rates. However, these procedures have direct or indirect costs linked to a longer time of the investigation, higher demands for documentation or patient and physician education. Our tool will enable to assess the benefits of each modification and put it into perspective by comparison with increased costs. An incremental cost-effectiveness ratio of 100’000 USD per LYG will determine, whether any modification should be recommended or advised against.

Our microsimulation has also several inherent limitations: i) Key parameters of the natural history of CRC are unknown. This is especially relevant for the adenoma dwell time, the time from the appearance of an adenoma until its transition to preclinical CRC. However, we provide three versions of CMOST spanning a wide range of reasonable assumptions for this parameter, enabling sensitivity analyses. ii) The serrated adenoma pathway is not explicitly considered by our model. While implementation of an additional adenoma path with preferential distribution of serrated adenoma lesions in the right colon and lower detectability by colonoscopy is technically feasible, most characteristics of serrated adenoma epidemiology remain unknown, reducing model calibration to guess work. However, our model allows for carcinoma directly developing from normal mucosa with a right-colonic preference implemented, at least partially accounting for the serrated adenoma path. iii) Our model does not account for benign non-adenomatous polyps. iv) CMOST has been calibrated to contemporary CRC incidence data. This contrasts previous models, which were calibrated using data of the year 1977, before the introduction of CRC screening. For these reasons, CRC incidence for MISCAN, SimCRC, and CRC-SPIN remains higher than for CMOST. Several studies indicate that the decrease in CRC incidence observed within the last decades is not only due to CRC screening but might also be due to usage of non-steroidal antirheumatic drugs [70] or changes in smoking [71] and nutrition [72]. These different effects cannot be disentangled.

## Conclusions

In summary, we have introduced CMOST, a new CRC microsimulation tool, freely available under a general public license. Predictions of our model regarding the natural history of CRC and CRC screening were similar to predictions of published CRC microsimulation tools and an endoscopic CRC screening study, confirming the validity of our model. CMOST predicts CRC screening by colonoscopy to be highly cost-efficient with an optimal time point of a single colonoscopy for maximum cost-efficiency of approximately 61 years. Our computations confirm adenoma dwell time as a critical parameter for CRC microsimulation models; however, similar results for various effects of colonoscopy screening including incidence reduction, cost-efficiency, and optimal time points were obtained with a dwell time of 8, 13, and 19 years.

Our tool enables assessment of many practical questions in current gastroenterology. For instance, cost-effectiveness of new CRC screening approaches, or of incremental changes of existing screening approaches such as improvements in colonoscopy screening by improved bowel preparation or physician training can be assessed. Additionally, cost-effectiveness of CRC screening at extremes of age, comorbidities or risks could be addressed. Basically, most practical question regarding screening or treatment of CRC which can be expressed in terms of risks and costs, our tool should be able to answer, perhaps after modification of the code. Thereby, the flexibility of our model enables adaptation also to countries with different CRC epidemiology such as in developing countries. Additional case studies will improve the practical value of our program in the future.

The main limitations of our tool is the limitation in our knowledge of CRC, making assumptions for adenoma dwell time and the distribution of risks for adenoma progression and initiation necessary. Furthermore, several aspects of the natural history of CRC such as the serrated adenoma pathway are not reflected by our program to limit complexity. Results of ongoing randomized CRC screening trials using colonoscopy (expected within 5-10 years) will improve the validity of predictions of our tool.

## Additional files

Additional file 1: Table S1. All versions of CMOST were calibrated relative to the 9 categories indicated below (105 data points). For parameters with gender differences (early and advanced adenoma prevalence, cancer incidence) the parameter for the whole population was adjusted with a correction factor for males and females. For stage distribution of symptomatic and asymptomatic cancer the distribution of time spent in a given stage as well as the stage at which the cancer would be symptomatic was adjusted to yield in a sojourn time of 3 years and the indicated stage distributions (benchmarks 8 and 9). (DOCX 32 kb)

Additional file 2: Table S7. Costs of colonoscopy and its complications. (DOCX 13 kb)

Additional file 3: Table S8. Stage dependent costs of cancer treatment. (DOCX 13 kb)

Additional file 4: Manual and Literature. (DOCX 40 kb)

Additional file 5: Table S9. Simulation of rectosigmoidoscopy screening for prevention of CRC according to randomized controlled trials (intention to treat analysis). Modeling of these studies provides additional validation of our model. (DOCX 19 kb)

Additional file 6: Table S2. Comparison of CMOST with other microsimulation models [64]: Individuals with and without preclinical disease at age 55 (adenomas, undiagnosed cancer) were identified and the cancer rates over the next 20 years were compared. (DOCX 13 kb)

Additional file 7: Table S3. Comparison of CMOST models with other microsimulation models [64]: For cancer diagnosed at the indicated ages (55, 65 or 75 years) the percentage of cancer developing over the last ≤10 years or ≤20 years before cancer diagnosis (i.e. adenomatous precursor present) is indicated. (DOCX 14 kb)

Additional file 8: Table S4. Comparison of CMOST models with other microsimulation models [64]: Adenoma dwell time cancer sojourn time and overall dwell time are indicated. Direct cancer was ignored for calculations of dwell time. (DOCX 14 kb)

Additional file 9: Table S5. Comparison of CMOST models with other microsimulation models [65]: Predicted incidence reduction of various screening interventions. (DOCX 13 kb)

Additional file 10: Table S6. Comparison of CMOST models with other microsimulation models [65]: Predicted mortality reduction of various screening interventions. (DOCX 13 kb)

## Abbreviations

CMOST: Colon modeling with open source tool
CRC: Colorectal cancer
CRC-SPIN: Colorectal cancer simulated population model for incidence and natural history
ICER: Incremental cost effectiveness ratio
LYG: Life years gained
MISCAN: Microsimulation screening analysis
SimCRC: Simulation model of colorectal cancer (SimCRC)
