# Supplementary material for: CMOST: an open-source framework for the microsimulation of colorectal cancer screening strategies
Source: BMC Med Inform Decis Mak. 2017 Jun 5;17:80. doi: 10.1186/s12911-017-0458-9 (PMC5460500; doi:10.1186/s12911-017-0458-9)
Supplement: Supplementary file 1 — All versions of CMOST were calibrated relative to the 9 categories indicated below (105 data points). For parameters with gender differences (early and advanced adenoma prevalence, cancer incidence) the parameter for the whole population was adjusted with a correction factor for males and females. For stage distribution of symptomatic and asymptomatic cancer the distribution of time spent in a given stage as well as the stage at which the cancer would be symptomatic was adjusted to yield in a sojourn time of 3 years and the indicated stage distributions (benchmarks 8 and 9). (DOCX 32 kb) [file 12911_2017_458_MOESM1_ESM.docx]

1. **BENCHMARKS**

Additional file 1: Table S1:

**Benchmark 1:** Early adenoma prevalence (percent of population with ≥1 adenoma) [1, 2]

| Age (years) | 25 | 35 | 45 | 60 | 70 | 80 |
| --- | --- | --- | --- | --- | --- | --- |
| Early adenomas, overall | 3% | 6% | 12% | 24% | 32% | 36% |
| Early adenomas, male | 3% | 8% | 15% | 30% | 39% | 45% |
| Early adenoma, female | 2% | 4% | 8% | 18% | 26% | 32% |

**Benchmark 2:** Advanced adenoma prevalence (percent of population with ≥1 advanced adenoma) [1, 3]

| Age (years) | 57 | 62 | 67 | 72 | 77 | 85 |
| --- | --- | --- | --- | --- | --- | --- |
| Advanced adenomas, overall | 4.8% | 5.8% | 6.6% | 7.7% | 8.1% | 8.4% |
| Advanced adenomas, male | 6.2% | 7,5% | 8.4% | 9.4% | 9.7% | 9.5% |
| Advanced adenoma, female | 3.4% | 4.2% | 4.8% | 5.8% | 6.5% | 7.3% |

**Benchmark 3:** Cancer incidence (per 100,000 per year) [4]

| Age (years) | 22 | 27 | 32 | 37 | 42 | 47 | 52 | 57 | 62 | 67 | 72 | 77 | 82 | 87 |
| --- | --- | --- | --- | --- | --- | --- | --- | --- | --- | --- | --- | --- | --- | --- |
| Incidence  overall | 4 | 7 | 12 | 23 | 33 | 46 | 65 | 103 | 135 | 180 | 222 | 264 | 270 | 343 |
| Incidence  male | 4 | 10 | 17 | 29 | 42 | 64 | 79 | 134 | 169 | 219 | 284 | 330 | 353 | 384 |
| Incidence  female | 5 | 4 | 7 | 17 | 24 | 29 | 51 | 74 | 103 | 145 | 170 | 212 | 211 | 313 |

**Benchmark 4:** Adenoma stage distribution [5, 6]

| Adenoma stage | I | II | III | IV | V | VI |
| --- | --- | --- | --- | --- | --- | --- |
| Percentage of all early adenomas | 38.4 | 29.7 | 23.1 | 8.7 |  |  |
| Percentage of all advanced adenomas |  |  |  |  | 79.8 | 20.2 |

**Benchmark 5:** Distribution of simultaneous multiple adenomas in a population of 54-74 year old individuals [7]

| Number of adenomas | 1 | 2 | 3 | 4 | ≥5 |
| --- | --- | --- | --- | --- | --- |
| Percentage of individuals with n adenomas | 27 | 13 | 7 | 4 | 3 |

**Benchmark 6:** Relative likelihood of an adenoma transforming to carcinoma [6, 8]

| Adenoma stage | I | II | III | IV | V | VI |
| --- | --- | --- | --- | --- | --- | --- |
| Relative likelihood of transformation | 0.002 | 0.21 | 0.3 | 1.06 | 12.8 | 85.5 |

**Benchmark 7:** Percentage of rectal carcinoma of all carcinoma [4]

| Rectal carcinoma percentage (age group) | 61-65 | 71-75 |
| --- | --- | --- |
| Percentage overall | 31.2 | 25.8 |
| Percentage male | 34.1 | 28.6 |
| Percentage female | 28.3 | 23.0 |

**Benchmark 8:** Stage distribution of symptomatic cancer (i.e. cancer detected due to symptoms) [9]

| Cancer stage | Stage I | Stage II | Stage III | Stage IV |
| --- | --- | --- | --- | --- |
| Percentage of all cancer | 15 | 35.6 | 27.9 | 21.5 |

**Benchmark 9:** Stage distribution of asymptomatic cancer (i.e. cancer detected during screening) [9, 10]

| Cancer stage | Stage I | Stage II | Stage III | Stage IV |
| --- | --- | --- | --- | --- |
| Percentage of all cancer | 39.5 | 34.7 | 17.3 | 8.5 |
