# Supplementary material for: CMOST: an open-source framework for the microsimulation of colorectal cancer screening strategies
Source: BMC Med Inform Decis Mak. 2017 Jun 5;17:80. doi: 10.1186/s12911-017-0458-9 (PMC5460500; doi:10.1186/s12911-017-0458-9)
Supplement: Supplementary file 2 — Costs of colonoscopy and its complications. (DOCX 13 kb) [file 12911_2017_458_MOESM2_ESM.docx]

**III. COSTS USED IN CMOST FOR COST-EFFICIENCY ANALYSIS**

Additional file 2: Table S7:

| **Colonoscopy** | Costs in USD |
| --- | --- |
| Diagnostic colonoscopy | 1,160 |
| Lesion removal colonoscopy | 1,409 |
| **Complications** | Costs in USD |
| Serosa burn | 8,060 |
| Perforation | 16,891 |
| Bleeding not requiring transfusion | 1,159 |
| Bleeding requiring transfusion | 6,715 |
