# Supplementary material for: CMOST: an open-source framework for the microsimulation of colorectal cancer screening strategies
Source: BMC Med Inform Decis Mak. 2017 Jun 5;17:80. doi: 10.1186/s12911-017-0458-9 (PMC5460500; doi:10.1186/s12911-017-0458-9)
Supplement: Supplementary file 3 — Stage dependent costs of cancer treatment. (DOCX 13 kb) [file 12911_2017_458_MOESM3_ESM.docx]

**III. COSTS USED IN CMOST FOR COST-EFFICIENCY ANALYSIS**

Additional file 3: Table S8:

| Cancer stage | First year costs (USD) | Continuing costs (USD) | Final year costs (USD) |
| --- | --- | --- | --- |
| Stage 1 | 19,179 | 263 | 47,876 |
| Stage 2 | 33,866 | 1,551 | 45,399 |
| Stage 3 | 39,367 | 1,551 | 44,460 |
| Stage 4 | 45,481 | 4,126 | 45,843 |
