# Supplementary material for: CMOST: an open-source framework for the microsimulation of colorectal cancer screening strategies
Source: BMC Med Inform Decis Mak. 2017 Jun 5;17:80. doi: 10.1186/s12911-017-0458-9 (PMC5460500; doi:10.1186/s12911-017-0458-9)
Supplement: Supplementary file 4 — Manual and Literature. (DOCX 40 kb) [file 12911_2017_458_MOESM4_ESM.docx]

**Additional file 4**

**V. STRATEGY FOR AUTOMATED PARAMETER CALIBRATION**

Due to the complexity of the model, parameter calibration was performed sequentially in four separate steps, estimating subsequently the following parameters:

1. Overall adenoma prevalence, adenoma prevalence for males and females, adenoma stage distribution as well as the probabilities of multiple adenomas.
2. Advanced adenoma prevalence, advanced adenoma prevalence for males and females and advanced adenoma stage distribution.
3. Colorectal cancer incidence, cancer incidence for males and females, percentage of rectal carcinoma and probabilities of direct transformation to colorectal cancer for each adenoma stage.
4. Direct cancer rates (i.e. cancer without adenomatous precursors)

Age-dependent parameters are modeled with specific functional forms: age dependence of new adenoma appearance risk is modeled as a sigmoidal function, whereas progression of early and advanced adenomas are modeled as Gaussian functions (details in Table 1, main text). Three scalar parameters define each of these functions and had to be estimated. Additional parameters were adjusted simultaneously (see below). To determine the parameters iteratively, an initial parameter estimation and a heuristic protocol for iteratively modifying the parameter estimates are required:

For the initial parameter estimation, we made use of the benchmarks of the respective step, as follows: For example, in step 1, adjusting adenoma prevalence uses the vector of age-dependent adenoma appearance. This vector is defined by a sigmoid function with parameters *a*_0_, *a*_1_ and *a*_2_ (compare Table 1 in main text). As a starting point for the optimization of *a*_0_, *a*_1_, *a*_2_ we fitted a sigmoid function to adenoma prevalence (one of the benchmarks for this step, see Supplementary Table 1, Benchmark 1) since adenoma prevalence also follows a sigmoid function. Empirically, we found this procedure to work well and also to lead to quick convergence of the parameters. However, parameter fitting also worked with other initial guesses as long as parameters describing a monotonously increasing sigmoid curve are used for the initial parameter estimation.

For the heuristic parameter adjustment, benchmarks for the age-dependent early adenoma prevalence, the age-dependent advanced adenoma prevalence and the age-dependent cancer incidence (Supplementary Table 1, Benchmarks 1, 2 or 3, respectively) were fit to the following equation: $\frac{{FitBenchmark}_{0}}{1+e^{-\left( {FitBenchmark}_{1}*y-FitBenchmark{}_{2} \right)}}$ .

Where *y* is the year of reference and *FitBenchmark* the parameter for the fit for the benchmark for age dependent early or advanced adenoma prevalence or cancer incidence. The output of CMOST calculated at every iteration regarding age-dependent early adenoma prevalence, the age-dependent advanced adenoma prevalence and the age-dependent cancer incidence were fit to an identical equation:$\frac{{FitOutput}_{0}}{1+e^{-\left( {FitOutput}_{1}*y-FitOutput{}_{2} \right)}}$ .

Where *y* is the year of reference and *FitOutput* the parameter for the fit for the output for age dependent early or advanced adenoma prevalence or cancer incidence of a given iteration. Use of *FitBenchmark* and *FitOutput* for parameter modification is detailed in the description of Step 1, Step 2 and Step 3 below.

To further improve accuracy of model calibration, we used the Matlab implementation of a Nelder-Mead algorithm [5] to fine-tune parameters of the sigmoidal or Gaussian functions.

At each stage, a relative error was defined as the sum of the root mean square deviation of the calculated output of CMOST (for instance, early adenoma prevalence) divided by the respective benchmark value:

$$\sum_{k=1}^{n} \left[ 1-\frac{\left( Predicted Early Adenoma Prevalence(age=y_{k} \right)}{\left( Benchmark for Early Adenoma Prevalence(age=y_{k} \right)} \right]^{1/2}$$

where *y_k_* is the year, and *n* the number of data points (i.e., years) available for this benchmark. This relative error was used for monitoring the convergence of the optimal model parameters.

*STEP 1: EARLY ADENOMAS*

During step 1 of our calibration procedure we adjusted parameters of CMOST to meet the following benchmarks: i) overall adenoma prevalence, ii) adenoma prevalence for males and females, iii) Probability of multiple adenomas (i.e. fraction of individuals with 1, 2, 3, 4 and ≥5 adenomas and iv) stage distribution of early adenoma (i.e. fraction of 3mm, 5mm, 7mm and 9mm adenomas. Relevant benchmarks are provided in Supplementary Table 1, Benchmark 1, 4 and 5.

*i) Overall adenoma prevalence:* The risk for the appearance of new adenomas with age (*y*) was modeled using the sigmoid function: $\frac{a_{0}}{1+e^{-\left( a_{1}y-a_{2} \right)}}$ . For the initial parameter estimation parameters *a*_0_, *a*_1_ and *a*_2_ were fitted to the early adenoma benchmark (see above) but similar final parameters were obtained by using different initial guesses (not shown). For the heuristic parameter adjustment, coefficients of the sigmoid curve were modified at every iteration as follows:

$${a_{0}\left( i \right) =a}_{0}\left( i-1 \right)\times exp \left( -0.1\times\left( {FitOutput}_{0}\left( i \right)-{FitBenchmark}_{0} \right) \right)\times\exp\left( -0.1\times{BenchmarkAdenomaDistribution}_{1}\div{CalculatedAdenomaDistribution}_{1}\left( i \right) \right)$$

$$a_{1}\left( i \right)=a_{1}\left( i-1 \right)-{FitOutput}_{1}\left( i \right)-{FitBenchmark}_{1}$$

$$a_{2}\left( i \right)=a_{2}\left( i-1 \right)-{0.5\times FitOutput}_{2}\left( i \right)-{FitBenchmark}_{2}$$

where *i* refers to the number of the current iteration, *a*_0_, *a*_1_ and *a*_2_ are the coefficients of the sigmoid function, *FitOutput*_0_, *FitOutput*_1_, *FitOutput*_2_ refer to coefficients of the sigmoid fit of the calculated adenoma prevalence (see above), *FitBenchmark*_0_, *FitBenchmark*_1_, *FitBenchmark*_2_ refer to coefficients of the sigmoid fit of the benchmark for adenoma prevalence, *BenchmarkAdenomaDistribution*_1_ refers to the benchmark for the fraction of individuals with 1 adenoma and *CalculatedAdenomaDistribution*_1_ refers to the calculated fraction of individuals with 1 adenoma for the indicated iteration.

*ii) Adenoma prevalence for males and females*: To account for different adenoma prevalence rates in males and females a scaling factor is applied to the new adenoma appearance rate. This parameter is adjusted using the deviations of calculated male and female adenoma prevalence rates at various ages to the benchmarks:

$${CF}_{female}\left( i \right)={CF}_{female}\left( i-1 \right)\times\sqrt{\frac{\sum_{y_{j=1}}^{6} \left( \frac{{CalculatedAdenomaPrevalence}_{male}\left( i,y_{j} \right)}{{BenchmarkAdenomaPrevalence}_{male}\left( y_{j} \right)} \right)}{\sum_{y_{j=1}}^{6} \left( \frac{{CalculatedAdenomaPrevalence}_{female}\left( i,y_{j} \right)}{{BenchmarkAdenomaPrevalence}_{female}\left( y_{j} \right)} \right)}}$$

where *CF_female_* is the correction factor for adenoma appearance rate for female gender, *CalculatedAdenomaPrevalence_male_*(*i,y_j_)* or *CalculatedAdenomaPrevalence_female_*(*i,y_j_*) the prevalence of adenomas at a given age, $y_{j=1:6}=\left[ 25, 35, 45, 60, 70, 80 \right] years,$ for the respective benchmark,

*BenchmarkAdenomaPrevalence_male_* or *BenchmarkAdenomaPrevalence_female_* the benchmark for prevalence of adenomas at a given age.

*iii) Probability of multiple adenomas*

The adenoma frequency distribution is indirectly adjusted by changing the Individual risk distribution: The risk of adenoma/cancer appearance in different individuals differs because of their genetic constitution, as well as lifestyles. This is reflected in the fraction of individuals with 1, 2, 3, 4 or ≥5 simultaneous adenomas. The individual risk distribution is a cumulative density function, describing the relationship between population proportions and individual risks for appearance of new adenomas. The function is defined by 0,0 and 5 additional anchor points and interpolated in between.

The risk levels of these 5 anchor points are iteratively corrected by comparing the output for the number of simultaneous adenomas with its corresponding benchmark:

$$\mathrm{Coeff}_{k}\left( i \right)=\mathrm{Coeff}_{k}\left( i-1 \right)\times\sqrt{\frac{{BenchmarkAdenomaDistribution}_{k}}{{CalculatedAdenomaDistribtution}_{k}(i)}}$$

Where *Coeff* refers to the anchor points of the curve describing the individual adenoma risk. This curve starts at (0,0) (i.e. the individual with the lowest risk has an adenoma risk of 0. *Coeff*_1_: anchor point at 20% of individuals with the lowest risk, *Coeff*_2_: anchor point at 86% of individuals, *Coeff*_3_: anchor point 94% of individuals

*Coeff*_4_: anchor point at 99% of individuals, *Coeff*_5_: anchor point at 100% (i.e. risk of the individual with the highest risk).

*BenchmarkAdenomaDistribution*_1…5_ refer to the Benchmark for the fraction of individuals with 1…≥5 adenomas, *CalculatedAdenomaDistribution*_1…5_: respective values calculated for iteration *i*.

*iv) Stage distribution of early adenomas*: The percentages of adenomas in stage I-IV from all early adenomas (i.e., 3, 5, 7 and 9mm size) were matched by adjusting the stage dependent adenoma progression risks of the immediate precursor of this adenoma (i.e. the previous state) according to the observed prevalence of the respective adenomas.

$${PR}_{stage}\left( i \right)={PR}_{stage}\left( i-1 \right) \times\sqrt[3]{\frac{{BenchmarkAdenomaStageDistribution}_{stage+1}}{{CalculatedAdenomaStageDistribution}_{stage+1}(i)}}$$

Where *PR_Stage_* refers to the stage specific adenoma progression rate, *Stage* to the stage of the adenoma (I…IV, i.e. 3mm…9mm), *BenchmarkAdenomaStageDistribution* the benchmark for the relative frequency of an adenoma stage relative to all early adenomas (Supplementary Table 1, Benchmark 4), *CalculatedAdenomaStageDistribution* the calculated value for the relative frequency of an adenoma stage relative to all early adenomas for a given iteration.

For optimal results this heuristic algorithm was used first, followed by the Nelder-Mead algorithm for adjusting *a*_0_, *a*_1_ and *a*_2_ as well as the five anchor points for the individual risk distribution (*Coeff*_1…5_).

*STEP 2: ADVANCED ADENOMAS*

During step 2, CMOST parameters were adjusted to meet the following benchmarks: i) overall advanced adenoma prevalence, ii) advanced adenoma prevalence for males and females, iii) advanced adenoma distribution (i.e. fraction of individuals with stage V and VI adenomas; >10mm and >20 mm adenomas, respectively). Relevant benchmarks are provided in Supplementary Table 1, Benchmark 2 and 4.

*i) Advanced adenoma prevalence:* To adjust CMOST parameters to meet this output the vector age dependent early adenoma progression rate was adjusted. Age dependent early adenoma progression with age (*y*) was modeled using as a Gaussian function: $b_{0}\exp\left( -\left( b_{1}y-b_{2} \right)^{2} \right)$. For the initial estimation of the parameters *b_0_, b_1_* and *b_2_* the function was fitted to the benchmark itself (*advanced* adenoma prevalence) but very similar results were obtained by using different initial estimations (not shown).

The parameters *b_0_, b_1_* and *b_2_* were adjusted iteratively using a heuristic algorithm such that the *advanced* adenoma distribution predicted by CMOST approaches its corresponding benchmarks:

$$b_{0}\left( i \right)=b_{0}\left( i-1 \right)\times\exp\left( -0.19\times\left( FitOutput_{0}\left( i \right)-FitBenchmark_{0} \right) \right)$$

$$b_{1}\left( i \right)=b_{1}\left( i-1 \right)-{0.1\times FitOutput}_{1}\left( i \right)-{FitBenchmark}_{1}$$

$$b_{2}\left( i \right)=b_{2}\left( i-1 \right)-{0.1\times FitOutput}_{2}\left( i \right)-{FitBenchmark}_{2}$$

where *i* refers to the number of the current iteration, *b*_0_, *b*_1_ and *b*_2_ are the coefficients of the Gaussian function for the age dependent early adenoma progression rate, *FitOutput*_0_, *FitOutput*_1_, *FitOutput*_2_ refer to coefficients of the fit of the calculated age dependent advanced adenoma prevalence, *FitBenchmark*_0_, *FitBenchmark*_1_, *FitBenchmark*_2_ refer to coefficients of the sigmoid fit of the benchmark for advanced adenoma prevalence (see strategy for automated parameter calibration, above).

*ii) Advanced adenoma prevalence for males and females*: To account for different advanced adenoma prevalence for males and females, a scaling factor for early adenoma progression for females was adjusted as described for Step 1.

*iii) Stage distribution of advanced adenomas*: The percentages of advanced adenoma stages V and VI (>1cm or advanced histology and >2cm, respectively) were matched by adjusting the adenoma progression of stage V adenomas according to the observed prevalence as described for Step 1.

As described for Step 1, this heuristic algorithm was applied first, followed by fine-tuning using the Nelder-Mead algorithm for parameters b_0_, b_1_, b_2_ and the progression rate for advanced adenoma stage V.

*STEP 3: CANCER INCIDENCE*

During Step 3 CMOST parameter adjustment is done to meet the following benchmarks: i) overall carcinoma incidence, ii) carcinoma incidence for males and females, iii) fraction of rectum carcinoma, iv) transformation rates for adenomas directly to cancer. Relevant benchmarks are provided in Supplementary Table 1, Benchmark 3, 6 and 7.

*i) Cancer incidence:* To calibrate cancer incidence the vector age dependent advanced adenoma progression is adjusted. This vector was modeled as a Gaussian function using y as age and parameters *c_0_, c_1_* and *c_2_* to be determined iteratively: $c_{0}\exp\left( -\left( c_{1}y-c_{2} \right)^{2} \right)$. An initial guess for the parameters *c*_0_, *c*_1_ and *c*_2_ was generated by fitting this function to the desired benchmark output itself (*cancer incidence*) but very similar final results were obtained using different initial guesses (not shown). The parameters *c*_0_, *c*_1_, *c*_2_ were heuristically altered at every iteration:

$$c_{0}\left( i \right)=c_{0}\left( i-1 \right)\times\exp\left( \frac{{-0.004\times FitAdvAdenomaBenchmark}_{0}}{{FitAdvAdenomaOutput}_{0}\left( i \right)}\times\left( FitOutput_{0}\left( i \right)-FitBenchmark_{0} \right) \right)$$

$$c_{1}\left( i \right)=c_{1}\left( i-1 \right)-{0.1\times FitOutput}_{1}\left( i \right)-{FitBenchmark}_{1}$$

$$c_{2}\left( i \right)=c_{2}\left( i-1 \right)-{0.5\times FitOutput}_{2}\left( i \right)-{FitBenchmark}_{2}$$

Where FitOutput_0_, FitOutput_1_, FitOutput_2_ and FitBenchmark_0_, FitBenchmark_1_, FitBenchmark_2_ refer to the fit for calculated and benchmarked carcinoma incidence, respectively (as explained in strategy for automated parameter calibration, above). FitAdvAdenomaBenchmark0 and FitAdvAdenomaOutput0 refer to the same fit for calculated and benchmarked advanced adenoma prevalence.

*ii) Carcinoma incidence for males and females*: As in Steps 1 and 2, a scaling factor for the age dependent advanced adenoma progression rate for females was adjusted to match gender specific carcinoma incidence rates.

*iii) Fraction of rectum carcinoma:* To meet this benchmark the location specific adenoma progression rate and advanced adenoma progression rate for the rectum (compare Figure 2, main manuscript) were adjusted.

$$CF_{rectum}\left( i \right)=CF_{rectum}\left( i-1 \right)\times\sqrt[3]{\frac{BenchmarkFractionRectumCa}{OutputFractionRectumCa(i)}}$$

Where *CF_rectum_* refers to the correction factor for early and advanced adenoma progression in the rectum, *BenchmarkFractionRectumCa* refers to the benchmark for the fraction of rectum carcinoma compared to all CRC, *OutputFractionRectumCa* to the calculated fraction of rectum carcinoma for iteration *i*.

*iv) Transformation rates for adenomas directly to cancer:* To meet benchmarks, stage dependent adenoma transformation rates were adjusted using calculated and assumed progression rates.

As described for early and advanced adenomas this heuristic algorithm for adjusting *c*_0_, *c*_1_ and *c*_2_ and the remaining parameters was used first, followed by fine-tuning using a Nelder-Mead algorithm adjusting *c*_0_, *c*_1_ and *c*_2._

*STEP 4 DIRECT CANCER*

A fraction of all cancers might arise without adenomatous precursors detectable by colonoscopy (i.e., direct cancer). This direct cancer pathway also accounts for serrated cancer with flat and hard-to-detect lesions. Direct cancer was assumed to arise preferentially in the right colon.

Direct cancer was adjusted indirectly by using results of a randomized rectosigmoidscopy study [6]. Direct cancer rates were stepwise increased with 25 repeated calculations with 100,000 individuals (to account for noise). CRC incidence reduction by rectosigmoidoscopy was linearly dependent on the direct cancer rate. The direct cancer rate predicted to yield in the overall incidence reduction of the Atkin study (23%) [6] was used. Direct cancer was censored at <5% of all cancers.

*STEP 2 and STEP 3 calibration*

Best agreement with advanced adenoma prevalence and cancer incidence benchmarks were obtained after using *b*_0_, *b*_1_ and *b*_2_ as well as *c*_0_, *c*_1_ and *c*_2_ as input for a subsequent calibration using the Nelder-Mead algorithm after Step 4.

*ADENOMA DWELL TIME*: The probability of progression of early and advanced adenomas is assumed to be low for most adenomas but high for a few adenomas, since a large fraction of adenomas will not progress at all, while a small fraction of adenomas confers high risk. While there is no direct guideline for the choice of this distribution, different alternatives result in different dwell times for the CMOST model. The input parameter sets for the different dwell time distributions (CMOST8, CMOST13, CMOST19) were manually adjusted and are provided along with the CMOST package.

**VI REFERENCES**

1. Kuntz KM, Lansdorp-Vogelaar I, Rutter CM, Knudsen AB, van Ballegooijen M, Savarino JE, Feuer EJ, Zauber AG: **A systematic comparison of microsimulation models of colorectal cancer: The role of assumptions about adenoma progression**. *Med Decis Mak* 2011, **31**(4):530-539.

2. Zauber AG, Lansdorp-Vogelaar I, Knudsen AB, Wilschut J, van Ballegooijen M, Kuntz KM: **Evaluating test strategies for colorectal cancer screening: a decision analysis for the U.S. Preventive Services Task Force**. *Ann Intern Med* 2008, **149**(9):659-669.

3. Holme O, Loberg M, Kalager M, Bretthauer M, Hernan MA, Aas E, Eide TJ, Skovlund E, Schneede J, Tveit KM *et al*: **Effect of flexible sigmoidoscopy screening on colorectal cancer incidence and mortality: a randomized clinical trial**. *Jama* 2014, **312**(6):606-615.

4. Segnan N, Armaroli P, Bonelli L, Risio M, Sciallero S, Zappa M, Andreoni B, Arrigoni A, Bisanti L, Casella C *et al*: **Once-only sigmoidoscopy in colorectal cancer screening: follow-up findings of the Italian Randomized Controlled Trial--SCORE**. *Journal of the National Cancer Institute* 2011, **103**(17):1310-1322.

5. Lagarias JC, Reeds JA, Wright MH, Wright PE: **Convergence Properties of the Nelder-Mead Simplex Method in Low Dimensions**. *SIAM Journal of Optimization* 1999, **9**(1):112-147.

6. Atkin WS, Edwards R, Kralj-Hans I, Wooldrage K, Hart AR, Northover JMA, Parkin DM, Wardle J, Duffy SW, Cuzick J *et al*: **Once-only flexible sigmoidoscopy screening in prevention of colorectal cancer: a multicentre randomised controlled trial**. *Lancet* 2010, **375**(9726):1624-1633.
