# Supplementary material for: CMOST: an open-source framework for the microsimulation of colorectal cancer screening strategies
Source: BMC Med Inform Decis Mak. 2017 Jun 5;17:80. doi: 10.1186/s12911-017-0458-9 (PMC5460500; doi:10.1186/s12911-017-0458-9)
Supplement: Supplementary file 5 — Simulation of rectosigmoidoscopy screening for prevention of CRC according to randomized controlled trials (intention to treat analysis). Modeling of these studies provides additional validation of our model. (DOCX 19 kb) [file 12911_2017_458_MOESM5_ESM.docx]

**III. Simulation of rectosigmoidoscopy screening**

Additional file 5 Table S9 :

|  |  | Holme et al. [3] with 95% CI | CMOST8 | CMOST13 | CMOST19 |
| --- | --- | --- | --- | --- | --- |
| Incidence reduction | All CRC | 20% (8-30%) | 30% | 27.9% | 26,7% |
|  | Right-sided CRC | 10% (-10 – 37) | 20% | 7.7% | 6.4% |
|  | Left-sided CRC | 24% (8-37%) | 33% | 35% | 35% |
| Mortality reduction |  | 27% (6-44) | 40% | 34% | 32% |

|  |  | Segnan et al. [4] with 95% CI | CMOST8 | CMOST13 | CMOST19 |
| --- | --- | --- | --- | --- | --- |
| Incidence reduction | All CRC | 18% (4-31%) | 15% | 14.5% | 15% |
|  | Right-sided CRC | 9% (-20-31%) | 8% | 3% | -1.7% |
|  | Left-sided CRC | 24% (6-38%) | 17% | 18.5% | 20% |
| Mortality reduction |  | 22% (-8-44%) | 19% | 19% | 19.5% |
