# Supplementary material for: CMOST: an open-source framework for the microsimulation of colorectal cancer screening strategies
Source: BMC Med Inform Decis Mak. 2017 Jun 5;17:80. doi: 10.1186/s12911-017-0458-9 (PMC5460500; doi:10.1186/s12911-017-0458-9)
Supplement: Supplementary file 6 — Comparison of CMOST with other microsimulation models [64]: Individuals with and without preclinical disease at age 55 (adenomas, undiagnosed cancer) were identified and the cancer rates over the next 20 years were compared. (DOCX 13 kb) [file 12911_2017_458_MOESM6_ESM.docx]

**II. COMPARISON OF CMOST PREDICTIONS WITH OTHER MICROSIMULATIONS**

Additional file 6: Table S2:

| **Model** | **20-year cancer rates of individuals with and without preclinical disease at age 55** |
| --- | --- |
| MISCAN | x7 |
| SimCRC | x29 |
| CRC-SPIN | x75 |
| CMOST8 | x10.5 |
| CMOST13 | x6.3 |
| CMOST19 | x6.2 |
