# Supplementary material for: CMOST: an open-source framework for the microsimulation of colorectal cancer screening strategies
Source: BMC Med Inform Decis Mak. 2017 Jun 5;17:80. doi: 10.1186/s12911-017-0458-9 (PMC5460500; doi:10.1186/s12911-017-0458-9)
Supplement: Supplementary file 7 — Comparison of CMOST models with other microsimulation models [64]: For cancer diagnosed at the indicated ages (55, 65 or 75 years) the percentage of cancer developing over the last ≤10 years or ≤20 years before cancer diagnosis (i.e. adenomatous precursor present) is indicated. (DOCX 14 kb) [file 12911_2017_458_MOESM7_ESM.docx]

**II. COMPARISON OF CMOST PREDICTIONS WITH OTHER MICROSIMULATIONS**

Additional file 7: Table S3:

|  | **MISCAN** | **CRC-SPIN** | **SimCRC** | **CMOST8** | **CMOST13** | **CMOST19** |
| --- | --- | --- | --- | --- | --- | --- |
| % of lesions developed within 10 years before cancer diagnosis | | | | | | |
| Age 55 | 72 | 3 | 10 | 55 | 48 | 46 |
| Age 65 | 67 | 4 | 9 | 53 | 46 | 44 |
| Age 75 | 62 | 4 | 9 | 49 | 44 | 45 |
| % of lesions developed within 20 years before cancer diagnosis | | | | | | |
| Age 55 |  |  |  | 86 | 80 | 70 |
| Age 65 | 94 | 24 | 39 | 85 | 78 | 68 |
| Age 75 | 92 | 25 | 37 | 77 | 73 | 65 |
