# Supplementary material for: CMOST: an open-source framework for the microsimulation of colorectal cancer screening strategies
Source: BMC Med Inform Decis Mak. 2017 Jun 5;17:80. doi: 10.1186/s12911-017-0458-9 (PMC5460500; doi:10.1186/s12911-017-0458-9)
Supplement: Supplementary file 8 — Comparison of CMOST models with other microsimulation models [64]: Adenoma dwell time cancer sojourn time and overall dwell time are indicated. Direct cancer was ignored for calculations of dwell time. (DOCX 14 kb) [file 12911_2017_458_MOESM8_ESM.docx]

**II. COMPARISON OF CMOST PREDICTIONS WITH OTHER MICROSIMULATIONS**

Additional 8: Table S4 :

|  | **MISCAN** | **CRC-SPIN** | **SimCRC** | **CMOST8** | **CMOST13** | **CMOST19** |
| --- | --- | --- | --- | --- | --- | --- |
| Time from adenoma incidence to preclinical cancer (adenoma dwell time) | | | | | | |
| Mean | 7.6 | 24.2 | 21.2 | 10.5 | 16 | 21.7 |
| Median | 6.0 | 23.0 | 19.0 | 7.5 | 13.5 | 19.5 |
| IQR | 2-11 | 16-31 | 12-29 | 2.75-14.5 | 7.5-21.8 | 12.5-28.3 |
| Time from preclinical cancer to cancer diagnosis (sojourn time) | | | | | | |
| Mean | 3.0 | 1.6 | 4.0 | 3.1 | 3.2 | 3.2 |
| Median | 2.0 | 2.0 | 3.0 | 3.2 | 3.3 | 3.2 |
| IQR | 1-4 | 1-2 | 2-5 | 2.5-3.75 | 2.5-3.75 | 2.5-3.75 |
| Time from adenoma incidence to cancer diagnosis (overall dwell time) | | | | | | |
| Mean | 10.6 | 25.8 | 25.2 | 13.3 | 19.2 | 24.9 |
| Median | 9.0 | 24.0 | 23.0 | 10.75 | 16.8 | 22.8 |
| IQR | 5-14 | 17-33 | 15-33 | 6-17.8 | 10.8-25 | 15.8-32 |
