# Supplementary material for: CMOST: an open-source framework for the microsimulation of colorectal cancer screening strategies
Source: BMC Med Inform Decis Mak. 2017 Jun 5;17:80. doi: 10.1186/s12911-017-0458-9 (PMC5460500; doi:10.1186/s12911-017-0458-9)
Supplement: Supplementary file 9 — Comparison of CMOST models with other microsimulation models [65]: Predicted incidence reduction of various screening interventions. (DOCX 13 kb) [file 12911_2017_458_MOESM9_ESM.docx]

**II. COMPARISON OF CMOST PREDICTIONS WITH OTHER MICROSIMULATIONS**

Additional file 9: Table S5:

| Incidence  reduction | MISCAN | SimCRC | CMOST8 | CMOST13 | CMOST19 |
| --- | --- | --- | --- | --- | --- |
| FOBT-Hemoccult II | 37% | 57% | 35% | 35% | 32% |
| FOBT-Sensa | 50% | 73% | 39% | 39% | 37% |
| FIT | 47% | 71% | 40% | 40% | 37% |
| Flexible Sigmoidoscopy | 47% | 59% | 44% | 43% | 41% |
