# Supplementary material for: CMOST: an open-source framework for the microsimulation of colorectal cancer screening strategies
Source: BMC Med Inform Decis Mak. 2017 Jun 5;17:80. doi: 10.1186/s12911-017-0458-9 (PMC5460500; doi:10.1186/s12911-017-0458-9)
Supplement: Supplementary file 10 — Comparison of CMOST models with other microsimulation models [65]: Predicted mortality reduction of various screening interventions. (DOCX 13 kb) [file 12911_2017_458_MOESM10_ESM.docx]

**II. COMPARISON OF CMOST PREDICTIONS WITH OTHER MICROSIMULATIONS**

Additional file 10: Table S6:

| Mortality reduction | MISCAN | SimCRC | CMOST8 | CMOST13 | CMOST19 |
| --- | --- | --- | --- | --- | --- |
| FOBT-Hemoccult II | 53% | 69% | 48% | 46% | 44.% |
| FOBT-Sensa | 66% | 81% | 49% | 47% | 45% |
| FIT | 65% | 80% | 52% | 49% | 48% |
| Flexible Sigmoidoscopy | 59% | 62% | 51% | 47% | 44% |
